# Comprehensive review on the positive and negative effects of various important regulators on male spermatogenesis and fertility

**DOI:** 10.3389/fnut.2022.1063510

**Published:** 2023-01-16

**Authors:** Hu-He Chao, Ye Zhang, Pei-Yu Dong, Sangiliyandi Gurunathan, Xi-Feng Zhang

**Affiliations:** ^1^Development Center for Medical Science and Technology, National Health Commission of the People's Republic of China, Beijing, China; ^2^Advanced Medical Research Institute, Shandong University, Jinan, Shandong, China; ^3^College of Veterinary Medicine, Qingdao Agricultural University, Qingdao, China

**Keywords:** spermatogenesis, reproductive health, sperm, nutrients, fertility

## Abstract

With the increasing global incidence of infertility, the influence of environmental factors, lifestyle habits, and nutrients on reproductive health has gradually attracted the attention of researchers. The quantity and quality of sperm play vital roles in male fertility, and both characteristics can be affected by external and internal factors. In this review, the potential role of genetic, environmental, and endocrine factors; nutrients and trace elements in male reproductive health, spermatozoa function, and fertility potency and the underlying mechanisms are considered to provide a theoretical basis for clinical treatment of infertility.

## Introduction

Infertility is a disease with psychological, economic, and medical consequences that can lead to trauma and stress. According to the World Health Organization and the International Committee for Monitoring Assisted Reproductive Technology, infertility is a reproductive system disease defined as the failure to achieve clinical pregnancy after 12 months or more of routine unprotected sex (1). An estimated 45% of infertility cases involve only men while 20% involve both women and men (2). Male infertility is mainly diagnosed *via* semen analysis, including determination of the sperm concentration/total number, motility, and morphology (3). It is characterized by several features, such as oligospermia, asthenozoospermia, and teratozoospermia. Several factors have been reported to cause male infertility, including genetic, environmental, and endocrine factors (4).

Genetic abnormalities can lead to spermatogenic disorders and account for 15–30% of male infertility (2, 5). Environmental factors, such as exposure to heavy metals, affect male sperm production and fertility. The oxidative stress induced by exposure to heavy metals is thought to affect male sperm production. Endocrine disruptor chemicals (EDCs) alter endocrine system processes and adversely affect the organism itself or its offspring (6). Semen quality is the major index of male fertility and is determined on the basis of various factors; semen quality and quantity vary according to age, disease, sexual activity, and diet. Environmental factors can affect semen quality by impairing spermatogenesis, steroid production, and sperm function, resulting in a decline in male fertility (7). Trace elements play a significant role in sexual health, spermatogenesis, sperm maturation, and capacitation. Human semen contains many trace elements, such as iron, zinc, copper, strontium, selenium, molybdenum, manganese, lead, arsenic, cadmium, vanadium, and cobalt. These elements are essential for normal spermatogenesis and sperm movement, maturation, and capacitation (8). Zinc, manganese, selenium, and copper have antioxidant effects and reduce lipid peroxidation (9). Magnesium and calcium maintain the osmotic balance of the male reproductive system and contribute to nutrient transport and gland function. The lack of some trace elements can also perturb reproductive function and sperm quality, which can negatively impact spermatogenesis, sperm quality, and male fertility (10).

Chemical elements play important roles in male reproduction because imbalance of micronutrients or macronutrients may lead to spermatogenesis defects and decreased libido, resulting in male infertility. Dietary and feed supplements can improve the male reproductive performance and help eliminate infertility. This review provides up-to-date information to better understand the positive and negative effects of selected macronutrients and micronutrients on male fertility.

To our knowledge, this is the first comprehensive review on the effects of nutrients on male infertility. We present the positive and negative effects of macronutrients and micronutrients; genetic, environmental, and endocrine factors; and trace elements on male spermatogenesis and fertility. We also discuss the conclusions and future perspectives on male infertility.

### Factors influencing spermatogenesis impairment and male infertility

Spermatogenesis is a complex differentiation process in which cells transform from spermatogonia to mature sperm through many stages and is a key event in male fertility. It is regulated by a myriad of factors that are involved in the development of subfertility. Several genetic abnormalities have been associated with impaired spermatogenesis. Abnormalities in chromosome structure and number have been found in 4% of patients with azoospermia or oligozoospermia, as an abnormal chromosome structure leads to abnormal meiosis and spermatogenesis failure (11). Around 4,000 genes have been reported to participate in human spermatogenesis. Genetic studies have revealed that mutations in the X chromosome and autosomal genes impair spermatogenesis, which has been reported in various animal models, including *Saccharomyces cerevisiae, Caenorhabditis elegans, Drosophila* (fruit fly), and *Mus musculus* (mouse) (12).

Sperm dysfunction is the most common cause of infertility in men. Male infertility is diagnosed on the basis of the presence of “oligozoospermia” (reduced sperm count), “asthenospermia” (decreased sperm motility), and “abnormal sperm” (sperm with an abnormal morphology). Oxidative stress leads to sperm dysfunction (13). The disorder related to the balance between oxidants and antioxidants may have a strong toxic effect on spermatogenesis through the production of excessive oxidants and male infertility (14). In humans, male infertility is closely associated with an increase in the exposure to heavy metals that affect semen and a decrease in the antioxidant content. It also results from increased lipid peroxidation induced by reactive oxygen species (ROS higher total plasma peroxidase activity) and the consequent antioxidant depression (lower total antioxidant capacity) observed in these individuals (13). In a previous study, high concentrations of iron and cadmium increased oxidative stress in the seminal plasma and sperm homogenate of the infertile group, decreased the content of antioxidants, and thus had a strong toxic effect on spermatogenesis by producing excessive oxidants and inducing apoptosis (14).

#### Genetic factors

Genetic factors play an important role in the most serious spermatogenic disorders, such as severe oligozoospermia or azoospermia (15). They cause ~15% of male infertility cases. Karyotype analysis has revealed that genetic factors control spermatogenesis. Chromosomal abnormalities, including deletion, inversion, mutation, aneuploidy, and translocation, are associated with male infertility, and many male infertility-related diseases are governed by various factors, such as chromosomal or monogenic diseases, mitochondrial DNA mutations, Y chromosome deletions, polygenic diseases, imprinted diseases, or hereditary endocrine diseases (16). Epidemiological evidence suggests that male infertility as a precursor for increased risk of diabetes, cardiovascular diseases, and all-cause mortality. Furthermore, studies have shown that abnormal semen parameters may also be linked to non-malignant diseases such as diabetes and ischaemic heart disease.

In infertile men, the incidence of chromosomal abnormalities has been reported to range from 5 to 15%, indicating that these abnormalities lead to non-obstructive azoospermia (NOA) (17). NOA affects about 1% of men in the general population and is characterized by clinical heterogeneity implying the involvement of several different acquired and genetic factors. Epidemiological evidence shows a link between azoospermia and higher morbidity and mortality rate, suggesting a common etiology for NOA and some chronic diseases, including cancer. Macroscopic deletions of the long arm of the Y chromosome can induce azoospermia. In a previous study, the rate of Y chromosome microdeletions in male patients with infertility averaged 8.2%, and the vast majority (84.3%) were associated with azoospermia (18). The azoospermia factor (AZF) region contains three different regions *AZF*a (792 kb), *AZF*b (6.2 Mb), and *AZF*c (4.5 Mb) which contain several genes responsible for male fertility (19). de Llanos et al. (20) reported that there is a partial deletion of 1.6 Mb on the Y chromosome, termed the “gr/gr” deletion, eventually leading to spermatogenic failure (20). Cytogenetic techniques have revealed the link between chromosomal abnormalities and male infertility owing to the presence of an extra X chromosome (47, XXY), which is characteristic of Klinefelter syndrome, and the X chromosome contains multiple genes and plays a significant role in the prophase of meiosis during mammalian spermatogenesis (21). Microdeletions affecting the AZF regions lead to phenotypic changes in infertile men, ranging from oligozoospermia to azoospermia in 2–10% of cases (22). Deletion of the *AZF*c region containing eight gene families causes progressive deterioration of spermatogenesis and, consequently, azoospermia eventually leads to phenotypic changes in infertile men (19, 22).

Spermatogenesis is thought to be regulated by as many as 2,000 genes, of which 600–900 genes seem to be expressed only in the male reproductive line. Approximately 10% of the genes in the genome are associated with spermatogenesis; some mutations in these genes may cause complete inhibition of spermatogenesis (5). There are many genetic factors related to male infertility, including single-gene and polygene defects. Tüttelmann et al. (23) reported that pathogenic mutations in male infertility genes, such as *NR5A1, DMRT1*, and *TEX11*, cause azoospermia in men (23).

Qualitative defects in spermatogenesis are characterized by various parameters, including sperm morphology, motility, and DNA and chromatin integrity, and by various conditions, including oligozoospermia, asthenozoospermia, teratozoospermia, and oligoasthenoteratozoospermia (OAT). Multiple morphological abnormalities of the sperm flagella (MMAF) include weakly malformed spermatozoa characterized by flagellum loss, shortness, curvature, curl, irregularity, and others. They are a mosaic with abnormal morphology and ultrastructural flagellar defects, such as central pair deletion, fibrous sheath dysplasia, double microtubule tissue disorder, or dynamic arm deletion. This suggests that the MMAF phenotype is genetically heterogeneous. Recently, several multiple MMAF pathogenic genes have been identified in humans (24, 25).

#### Endocrine factors

The endocrine system maintains the balance of the entire body through hormones. Testosterone and sperm are important products of testicular synthesis. Testosterone is necessary for the development and maintenance of several physiological functions. Testicular function depends on the regulation of hormones secreted by the hypothalamus, pituitary gland, and local endocrine and paracrine pathways. Spermatogenesis involves the synergistic action of endocrine hormones, many paracrine and growth factors, closely coordinated gene and protein expression programs, and genomes and epigenetics of different non-coding RNA species (26). Luteinising hormone (LH) and follicle-stimulating hormone (FSH) are the key endocrine factors controlling testicular function and spermatogenesis and are the main hormone regulators of spermatogenesis (27). In particular, LH stimulates high concentrations of testosterone (ITT) in the testis, which is essential for spermatogenesis. Germ cells are in close contact with Sertoli cells at all stages of spermatogenesis and require structural and functional support from Sertoli cells. Unlike germ cells, these somatic cells express sex steroids and FSH receptors, which are the most important hormones that regulate spermatogenesis. The increase in FSH signaling may be sufficient to initiate spermatogenesis by activating Sertoli cell function (28).

An imbalance in serum hormone levels causes disturbances in spermatogenesis and male infertility. For example, prolactin (PRL) is a polypeptide hormone, which is primarily secreted by lactotropic cells of the pituitary, which controls the production of LH and FSH *via* the regulation of gonadotropin-releasing hormone (GnRH) through feedback mechanism on the hypothalamus (29, 30). PRL is involved in various types of functions including lactation, osmoregulation, immune articulation and reproduction (31). FSH balance is regulated by the pituitary. If the sperm-producing potential of the testis decreases, then the pituitary starts producing more FSH to revive normal testicular function (32). Conversely, higher levels of FSH lead to azoospermia and severe-oligozoospermia, which are hallmarks for inactive or damaged seminiferous tubules (33). Studies suggest that increased serum PRL levels are associated with infertility, impotence, and hypogonadism (34, 35). A considerable amount of evidence suggests that several animal and human studies have shown that PRL positively modulates various aspects in testicular function hinting at a crucial role in male reproduction. PRL release is regulated by a wide variety of factors secreted from the hypothalamic pituitary gonadal (HPG) axis (29, 30). PLR receptors (PLR) appear to regulate of sertoli cell (36). High PRL levels lead to hypogonadism and male infertility (37). FSH, LH, thyroid stimulating hormone (TSH), and chorionic gonadotropin (hCG) are known to perform important functions such as Sertoli cell development. Exogenous application of testosterone reduces intra-testicular testosterone and spermatogenesis with consequent oligospermia and sometimes azoospermia (38, 39). Excessive levels of exogenous androgen cause temporary spermatogenic dysfunction, thus aggravating infertility (40). Exogenous testosterone is known for its contraceptive effects in men. Exogenous administration of androgens influences the HPG axis with negative feedback that may lead to a partial or complete cessation of spermatogenesis by decreasing FSH and LH (41). Exogenous testosterone ameliorates hypogonadism in males; conversely, it reduces sperm production and count. An et al. (42) reported that the effect of exogenous testosterone supplementation on spermatogenesis in a rat model of oligoasthenospermia (42). The rats were randomized equally into four groups: control, model, low dose, and high dose. After injection of testosterone undecanoate (TU), body weights, serum reproductive hormone levels, sperm measurements in the epididymis, and testis histology were monitored. The TU injections increased serum testosterone levels steadily. In addition, exogenous testosterone supplemental therapy (TST) increased the intra-testicular testosterone concentration modestly and alleviated the testicular oxidative stress markers. There was a significant improvement in sperm and testicular function due to decreased mitochondrial apoptosis in the testis by modulation of Bcl-2, Bax, caspase-3, and caspase-9 expression. Despite the positive effects of testosterone on sexual function, it has a negative effect on fertility. Exogenous testosterone therapy can negatively affect the HPG axis and inhibit the production of FSH and LH. Therefore, the usage of testosterone remains controversial (43).

#### Environmental factors and pollutants

Generally, humans are exposed to various hazardous chemicals including carcinogens; toxins; reproductive toxins; irritants; corrosives; neurotoxins; hepatotoxins; and chemicals that damage the lungs, skin, eyes, or mucous membranes. These hazardous chemicals enter human bodies through inhalation, ingestion, injection, and/or absorption through the skin and eyes. The toxic substances potentially damage biological systems, disturbing the functioning of biochemical processes and resulting in detrimental and even fatal effects. Owing to rapid urbanization and the development of chemical industries, numerous xenobiotics have been released into the environment. Among the various systems in the human body, the male reproductive system is highly sensitive to the environmental factors that contribute to infertility (44). The released chemicals mimic estrogen and EDCs and are possible factors responsible for male infertility. Environmental factors, such as living in pollution-enriched urban areas, influence sperm morphological defects and DNA fragmentation and increase the incidence of pear-shaped, slender, round heads; acrosome abnormalities; vacuolar chromatin; asymmetric neck; and ERC (45). Environmental pollutants, such as non-essential heavy metals, lead, cadmium, arsenic, mercury, and barium, can have harmful effects on semen and sperm quality.

EDCs can alter endocrine system processes and adversely affect the organism itself or its offspring. They have oestrogenic, anti-oestrogenic, androgen, or anti-androgen effects, can disrupt the hormonal system, and cause male reproductive dysfunction through a variety of mechanisms (46). Exposure to increasing EDC concentrations leads to decreased semen quality, which increases the incidence of male infertility and impairs male reproductive health (47). Bisphenol A (BPA) is a monomer used to make a wide variety of polycarbonate plastics and resins, which has oestrogenic activity and binds to the α-estrogen receptor (ER-α) and, to a lesser extent, the β-estrogen receptor (ER-β). It reduces serum FSH, LH, and testosterone concentrations. BPA increases the risk of cryptorchidism and azoospermia, decreases semen quality, increases the risk of testicular cancer, and reduces paternal fertility rates (48). Further, a mouse study revealed that BPA exposure leads to a decrease in normal sperm morphology and motility. Men exposed to dioxins exhibit high concentrations of morphologically abnormal sperm and low linear motility. Exposure to polychlorinated biphenyls and phthalates decreases sperm count and motility and alters normal morphology (49). Furthermore, exposure to pesticides alters normal sperm morphology and reduces sperm count, volume, and motility (49). Pesticides affect many organs, including the reproductive system, and related processes and parameters, including spermatogenesis, sperm density, testicular weight, motility, sperm count, sperm survival rate, and other sperm parameters. In particular, they induce sperm DNA damage and abnormal sperm morphology. Pesticides affect the male reproductive system by reducing the testis weight and impairing the function of the seminal vesicles, epididymis, and ventral prostate, leading to degeneration of the seminiferous tubules and changes in the concentrations of hormones, such as testosterone, FSH, and LH. They also affect the antioxidant enzymes in the testes and inhibit testicular steroid production.

Heavy metals refer to any metal chemical elements with a high density and that are toxic at low concentrations. These include mercury, arsenic, cadmium, chromium, lead, and thallium. Heavy metals have bioaccumulation properties, can enter the water supply system through industrial and domestic wastes, and even destroy the soil. They are released through acid rain into streams, rivers, lakes, and groundwater. Different environmental and occupational factors can affect male reproductive function. The adverse effects of exposure to heavy metals on the male reproductive system may be attributed to the disruption of the hypothalamic–pituitary axis or a direct influence on spermatogenesis, resulting in the deterioration of semen quality. There is a correlation between exposure to heavy metals and reduced semen quality. Moderate or high concentrations of lead can reduce sperm count, density, and motility, resulting in fertility problems in humans (50). Exposure of men to high concentrations of cadmium in seminal plasma (65 μg/dL) results in decreased sperm count and activity. Increasing concentrations of mercury in the blood (40.6 mmol/L) lead to harmful effects on sperm and cause a progressive motility rate of < 50% and a normal morphology rate of < 14% (51).

Heavy metal exposure is a potentially harmful factor in male sperm production and fertility (6). Heavy metals have toxic effects on reproductive processes and hormonal regulation. They are endocrine disruptors that interfere with the regulation of endogenous hormones and negatively impact endocrine function (52). The concentrations of heavy metals can be measured in the serum, semen, seminal plasma, and hair, but especially in the blood or urine (52). Among the various types of heavy metals, cadmium, arsenic, and lead are considered to be the major toxins affecting reproductive function.

Heavy metals cause toxicity by inducing oxidative stress, inflammation, endocrine disruption, and signaling pathway disruption (53). Cadmium, mercury, and lead are associated with Sertoli cell dysfunction. Toxicants affecting interstitial cells can lead to abnormal testosterone secretion and seminiferous epithelium reduction, resulting in impaired Sertoli cell function and spermatogenic dysfunction. The main cause of spermatogenesis failure is a change in the adhesion between germ and stem cells (54). Toxins interact with the secretion of LH and FSH, both of which play an important role in sperm quality.

**Figure 1:**
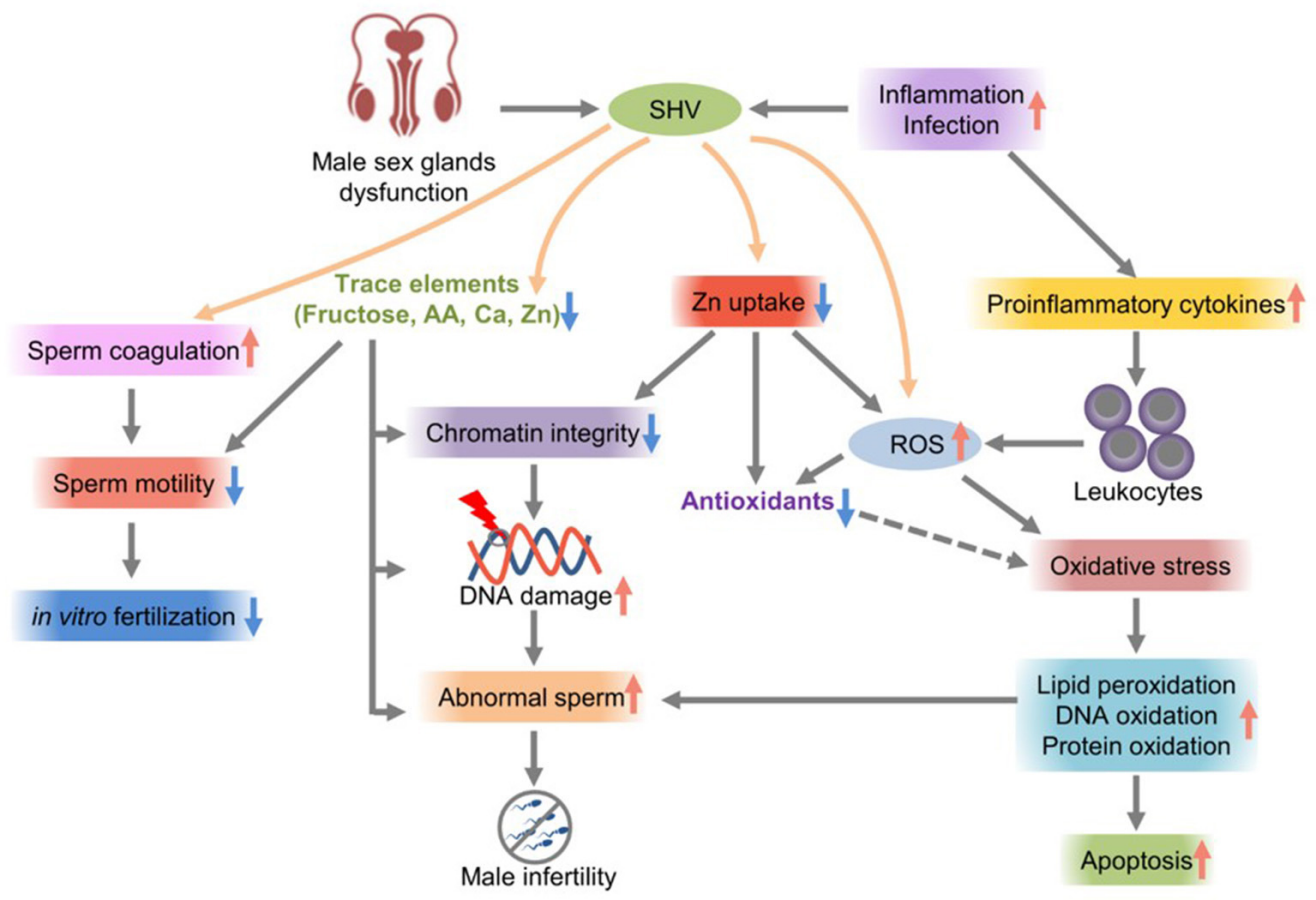
The effects of SHV on male infertility.

Lead and cadmium are endocrine disruptors that can change male hormone concentrations, leading to impaired semen quality and male infertility (55). Copper sulfate and cadmium chloride significantly reduce sperm motility parameters (56). A high semen viscosity is an important factor leading to poor sperm quality. Sperm motility is impaired; zinc uptake is insufficient, the trace element concentrations change, the semen quality is poor, the leukocyte count increases, and ROS production increases. Oxidative stress leads to imbalances in seminal plasma antioxidants, which is the main mechanism of a high semen viscosity, resulting in sperm DNA damage, sperm chromatin instability, membrane lipid peroxidation, and a low fertilization rate (Figure 1) (58).

Heavy metals found in seminal plasma can also increase the risk of poor sperm survival and abnormal sperm morphology (teratozoospermia) and ultimately affect male fertility by inducing ROS production, resulting in lipid peroxidation and sperm DNA damage (59). Oxidative stress plays an important role in human reproduction. Excessive ROS levels induce deleterious effects on human spermatozoa and damage the plasma membrane of spermatozoa, eventually leading to sperm dysfunction. ROS have potential significance in reproductive biology and play important physiological and pathological roles in male infertility (60). The physiological level of ROS is crucial for sperm maturation, capacitation, acrosome reaction, and fertilization. In fertile and infertile groups, hydrogen peroxide has harmful, dose-dependent effects on sperm DNA integrity and function. ROS regulate sperm chromatin condensation by controlling the number of germ cells and by inducing sperm apoptosis or proliferation (61). They are also involved in the capacitation, acrosome reaction, mitochondrial stabilization, and motility of mature sperm. ROS modify the integrity of DNA in the nucleus of 15 variants by inducing DNA strand breaks, base modification, and chromatin cross-linking (62). They are essential for reproductive function; however, increasing levels of ROS also have a detrimental effect on fertility.

## Effects of nutrients on male spermatogenesis and sperm quality

### Effect of diet on male spermatogenesis and sperm quality

**Figure 2:**
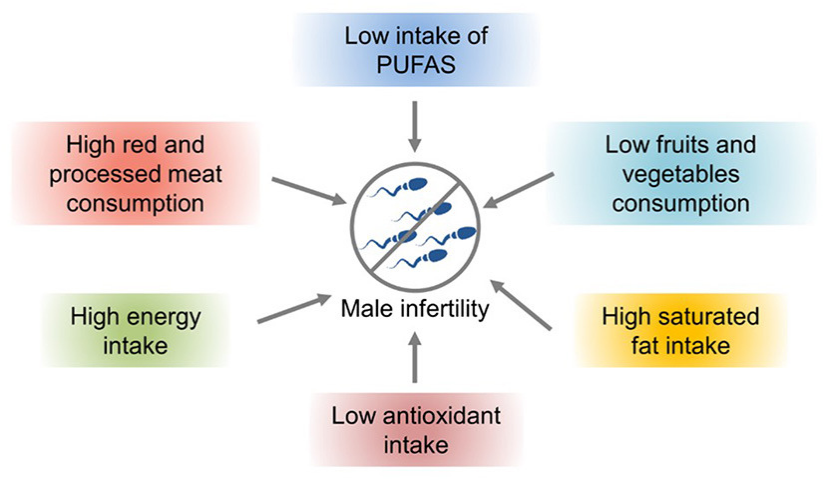
The effects of nutrients and lifestyle factors on male infertility (57).

Nutrients and lifestyle habits play significant roles in reproductive processes/conditions, particularly in male infertility. A hypercaloric diet and foods with low nutritional density have been associated with poorer semen parameters and lower fertility rates (Figure 2). Studies have shown that increased intake of trans-omega-6 fatty acids and decreased intake of omega-4 fatty acids are related to deterioration of testicular endocrine function (63). Intake of saturated fat can lead to decreased sperm density and semen count. For example, caffeinated sweet drinks are associated with reduced semen volume, sperm count, and sperm density (64). Improper diet and obesity can lead to lower semen quality and increase the risk of infertility. The intestinal microbiota plays a significant role in human health. However, an imbalance in the microbiota can lead to various complications, such as immunodeficiencies and a predisposition to metabolic diseases (65). Intake of large amounts of fat and monosaccharides may lead to intestinal biological disorders, resulting in increased intestinal barrier permeability and ultimately poor sperm quality and impaired spermatogenesis (66).

**Figure 3:**
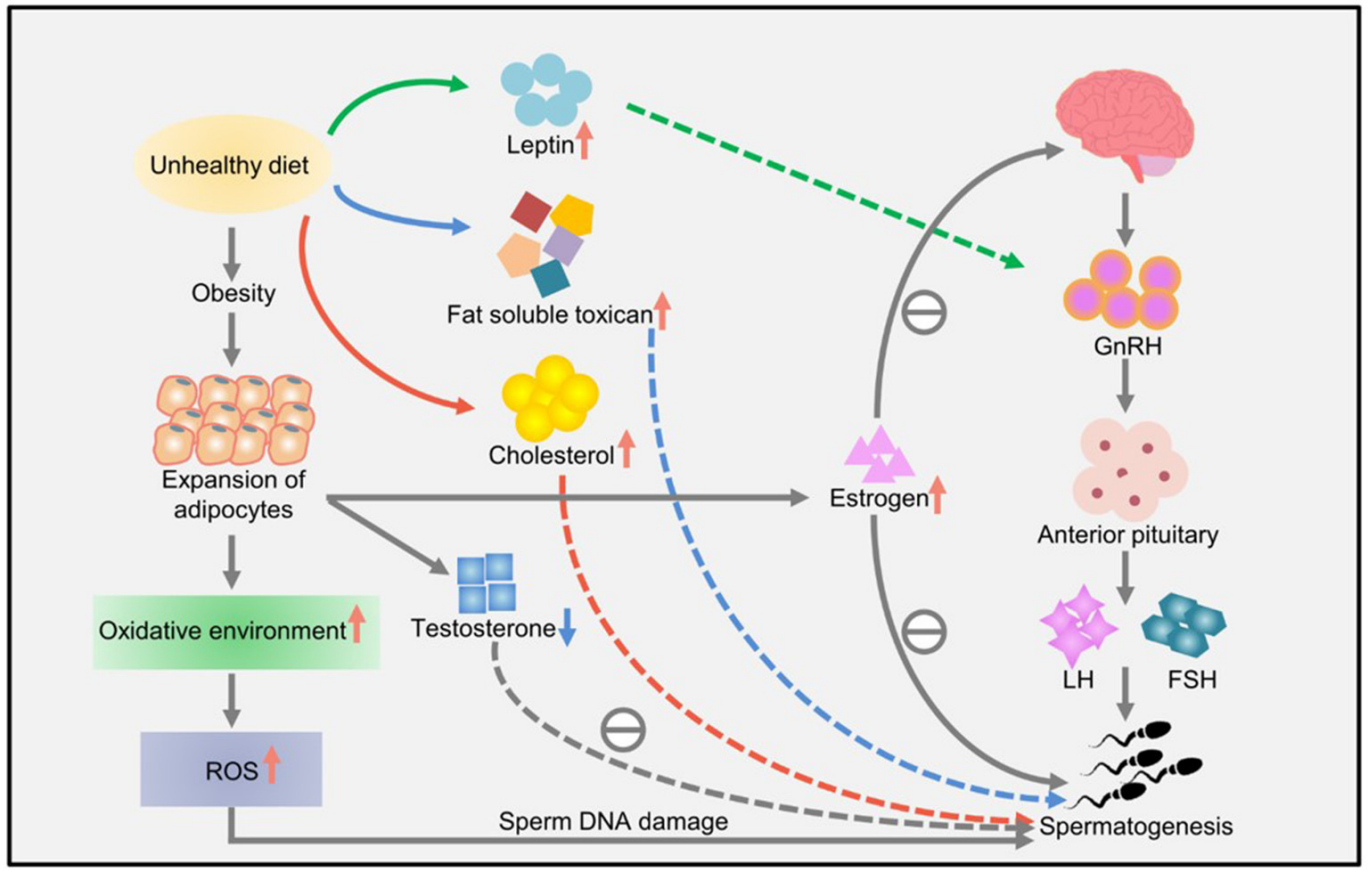
The effects of diet and obesity on spermatogenesis (57).

Lifestyle habits, such as smoking and drinking, can also reduce sperm quality. Studies have shown that obesity and a high-fat diet can lead to impaired reproductive ability, affecting the molecular sperm structure, fetal development, and offspring health (57). High-energy diets, such those with high amounts of trans fat and saturated fat, may affect fat metabolism in the testes, impair spermatogenesis, and alter sperm quality (67). Increased ROS levels have a harmful effect on sperm DNA, resulting in the formation of 8-oxydeoxyguanosine. Excessive consumption of high-calorie foods rich in fatty acids and trans fat leads to the accumulation of fatty acids and liposoluble toxins in the testicular environment, affecting spermatogenesis and testosterone production in Leydig cells (57). Owing to the lack of gonadotropin support, accumulation of a large amount of leptin leads to spermatogenesis disorders and infertility and consequently to a decline in sperm quality (57). A high-cholesterol diet may cause male infertility and reduce semen quality by destroying the blood–testicular barrier (67). The effects of diet and obesity on spermatogenesis are shown in Figure 3.

### Effect of lifestyle on male spermatogenesis and sperm quality

At present, lifestyle-related factors such as smoking, alcohol consumption, and use of mobile phones and computers are playing a significant role in male infertility. Studies have shown that sperm quality is essentially determined by obesity, nicotine addiction, heavy exposure to electromagnetic radiation emitting devices and alcohol consumption (68). The immense usage of mobile phones and other devises as a source of low-level radio-frequency electromagnetic fields (RF-EMF) causes poor semen quality. In particular, low-level RF-EMFexerts non-thermal and thermal effects on sperm quality. The non-thermal effect is believed to increase the production of reactive oxygen species, resulting in DNA damage (69, 70). The thermal effect causes testicular dysfunction because the device is carried in trouser pockets near external male reproductive organs; it might augment sperm DNA fragmentation (71). Studies have demonstrated that exposure of human spermatozoa to a laptop connected to the internet *via* Wi-Fi significantly decreases progressive semen motility and increases semen DNA fragmentation (72). Impaired sperm motility might be the results of EMF induced oxidation of phospholipids and high seminal ROS levels (73, 74). An *in vivo* study indicated that long lasting exposure to 4G LTE based EMF had deleterious effects on rat spermatogenesis (75).

Among all Word Health Organization regions, ~ 37% of men of reproductive age use tobacco (76). Cigarette smoke contains several hazardous substances, all of which exert harmful effects on male germ cells. Smoking cigarettes has been correlated with reduced semen quality, with impaired motility, decreased concentration, and abnormal morphology (77). Sepaniak et al. reported that there is a direct correlation between cigarette smoking and abnormal sperm chromatin (78). Aboulmaouahib et al. reported significant increases in the DNA fragmentation index and spermatic chromatin decondensation in smokers as well as smokers who consume alcohol (79). Further, a study provided evidence that long-time cigarette smoking leads to increased testosterone metabolism in the liver, concurrently causing secretory dysfunction of Leydig and Sertoli cells (80).

Alcohol consumption is the vital factor that decreases semen quality. Researchers have found a significant correlation between alcohol intake, semen volume, and sperm morphology and motility (81, 82). Another study found more pronounced hormonal changes in infertile patients who drink daily compared with the group of occasional drinkers (83). Alcohol consumption shows a significant impact on semen quality in male offspring and induces DNA damage in male germ cells (84).

### Effect of micronutrients on male spermatogenesis and sperm quality

Micronutrients are essential for proper bodily function; therefore, they are necessary in daily food intake. However, excess levels of micronutrients lead to harmful effects. Vitamins are organic micronutrients and play essential roles in the human body (85). Vitamin C is an electron donor vitamin capable of reducing metals and regenerating vitamin E from its oxidized form. Vitamin C prevents agglutination and protects against DNA damage caused by ROS in sperm cells (86). Vitamin E can protect cell membranes and prevents lipid peroxidation (87). Vitamin E has multiple functions in male fertility, such as testosterone biosynthesis and modulation of telomerase activity. An *in vivo* study demonstrated that in rats subjected to noise-generated stress and exposed to nicotine, vitamin E ameliorates the loss of sperm viability and increases fertilization rates (88, 89). Vitamin B9 is a water soluble compound and essential for DNA metabolism; it also protects DNA from mutations and strand breaks (90, 91). A study revealed that supplementing 5 mg/day of folic acid increases the sperm concentration and the normal sperm count (92).

## Trace elements ameliorate impaired male spermatogenesis and reduction in sperm quality

Human semen contains calcium, magnesium, zinc, selenium, copper, manganese, and other trace elements. These substances are essential for sexual health; spermatogenesis; and sperm maturation, motility, capacitation, and function. Deficiency in these trace elements negatively influences spermatogenesis and is responsible for poor sperm quality and low fertility rates in men (8).

Several micronutrients play significant roles in spermatogenesis. Sperm quality is regulated by various nutrients, including zinc, folate, selenium, and vitamins (93). Among these micronutrients, zinc plays multiple roles in the male reproductive system. It is considered a vital trace element for healthy growth, good neurological system function, and normal immune system responses (94).

### Zinc

Zinc is an essential micronutrient involved in the electron transfer reactions of a variety of enzymes in the human body (95). It participates in the anatomical development and normal function of the male reproductive system and promotes spermatogenesis by actively participating in the maturation of sperm and the preservation of the germinating epithelium. Low zinc concentrations can delay testicular development and lead to cessation of spermatogenesis. Zinc deficiency leads to a decrease in sperm count; this condition works only at the testicular level, leading to spermatogenic disorders and gonadal dysfunction, such as reduced testicular weight and seminiferous tubule atrophy (96). Zinc supplementation has a protective effect against lead damage, leading to degenerative changes in mature sperm. Zinc is a regulator of semen enzyme activity; it helps stabilize the chromatin and cell membranes of sperm and improves normal flagella, mid-segment formation, and sperm motility (97). Zinc and selenium deficiency in the blood has been linked to poor sperm quality. Huang et al. reported that zinc and selenium counteract cadmium-induced damage, including poor sperm quality and low sperm count (98). Zinc is an important nutrient and essential mineral in human semen. It is necessary for testicular development, normal testicular function, and epididymal and prostate function (99). For example, fertile individuals have significantly higher concentrations of zinc than infertile individuals (8). Zinc deficiency impairs spermatogenesis and lead to abnormal sperm quality (100). It reduces sperm quality through a variety of mechanisms, such as sex hormone deficiency, DNA damage, oxidative stress, apoptosis, spermatogenesis disturbance, and zinc finger protein deficiency. Meanwhile, zinc deficiency leads to male infertility through a variety of mechanisms, including testicular retardation, spermatogenesis disorders, sex hormone deficiency, oxidative stress, inflammation, and cell apoptosis. Furthermore, delayed sexual maturation, hypogonadism, testicular weight loss, Leydig cell damage, testicular atrophy, and seminiferous tubule damage can also occur (101). Zinc plays a significant role in the biosynthesis, storage, and secretion of male sex hormones, such as testosterone; its deficiency induces Leydig cell damage or apoptosis, which is associated with testosterone biosynthesis defects and spermatogenesis failure. Steroidogenesis can also be impaired, including decreased testosterone and progesterone production and increased LH and FSH production (102). Zinc is an important factor affecting sperm formation and regulates DNA replication, transcription, and packaging, as well as protein biosynthesis, cell differentiation, and proliferation. Deficiency in this element can lead to poor sperm quality and male infertility by increasing oxidative stress (8). High levels of oxidative stress, especially lipid peroxidation, have negative effects on spermatogenesis, sperm membrane fluidity, sperm capacitation, the sperm–egg interaction, and sperm transport during female reproduction and fertilization (103). These results indicate that zinc plays an important role in spermatogenesis and development.

Folate is an essential micronutrient required for germ cell development. The antioxidant properties of folate enhance sperm production and count and improve male sterility by inhibiting apoptosis and protecting against DNA damage (104). Vitamin E plays an important role in the survival of the male reproductive organs and sperm cells. It promotes the development of the reproductive organs by increasing epididymal weight, epididymal duct and seminiferous tubule diameter, and spermatogenic cell count and density, which are essential for the smooth progression of spermatogenesis. Vitamin E deficiency can lead to testicular abnormalities, spermatogenesis disorders, epididymal epithelial cell differentiation, incomplete spermatogenesis, and sperm cell degeneration (105). Vitamin C intake is closely associated with sperm concentration, count, and motility. A vitamin-free diet reduces testicular weight, male sex hormone production, and sperm quality (106).

### Calcium

Calcium acts as an intracellular messenger and participates in a variety of cellular functions, including sperm motility, hyperactivation, capacitation, the acrosome reaction, and chemotaxis in the female reproductive tract (107). Calcium deficiency impairs sperm motility: it is significantly lower in affected men than in healthy men. Further, calcium regulates spermatogenesis as well as the growth, differentiation, and proliferation of spermatogonia and spermatocytes (108). The seminal plasma calcium concentration is lower in infertile men with oligospermia, azoospermia, or oligoasthenospermia than in healthy men. Furthermore, in the presence of calcium, ATP facilitates flagellar movement and sperm motility (109). Beigi Harchegani et al. reported that calcium deficiency leads to male infertility by activating various mechanisms, such as spermatogenesis failure, impaired steroidogenesis, chemotactic failure, and impaired sperm motility, capacitation, and acrosome reaction (110). Calcium is involved in the induction of steroidogenesis and regulation of high testosterone production in Leydig cells. Collectively, these findings suggest that seminal plasma calcium is associated with male fertility and that lower concentrations lead to male infertility.

### Sodium and potassium

Sodium and potassium play significant roles in normal sperm function, semen production, and sperm capacitation. They are directly associated with spermatozoa motility and fertilization capacity. Low concentrations of sodium and potassium cause infertility; this is usually observed under normozoospermic, oligozoospermic, oligoasthenozoospermic, asthenozoospermic, and azoospermic conditions (111). A study suggested that male mice lacking sodium/potassium-ATPase were unable to fertilize eggs *in vitro* (112). Sodium and potassium regulate sperm membrane potential and fertilizing ability and play essential roles in sperm capacitation and testosterone production (111). Deficiency in these elements can lead to spermatogenesis disorders and fertility problems and abnormal progesterone concentrations and acrosome reactions in human sperm, respectively (111). Taken together, these data indicate the importance of sodium and potassium in sperm activity.

### Magnesium

Magnesium is an important trace element and activator of many enzymes in the phosphorus transfer reaction; it is involved in spermatogenesis and affects sperm motility (113). Lower concentrations of magnesium are associated with increased nitric oxide production and vasoconstriction, which may lead to premature ejaculation (114). Generally, infertile patients have lower concentrations of magnesium than fertile individuals. This element is directly associated with calcium and plays a significant role in semen transport, sperm motility, and male fertility (114). The energy required for sperm motility is released by magnesium-dependent ATPase (112). Collectively, low magnesium concentrations in seminal plasma play a significant role in infertility.

### Selenium

Selenium is an important element for maintaining male fertility. Selenium deficiency leads to increased oxidative stress and has a negative effect on spermatogenesis (115). This element regulates spermatogenesis and is mainly mediated by two selenoproteins, which provide an important link with sperm quality and male fertility (115). It significantly activates the secretion of testosterone and consequently induces spermatogenesis, and its concentrations are closely related to the quantity, vitality, and normal morphology of sperm. Morbat et al. reported that fertile men have higher selenium concentration than in infertile men (116). Lower selenium concentrations are associated with poor sperm quality and an increased risk of male infertility. Selenium has known antioxidant and beneficial effects: it protects sperm against oxidative stress induced DNA damage, which consequently enhances sperm motility and viability (117). Moreover, selenium supplementation significantly improves male fertility. In a previous study, selenium supplementation enhanced the concentrations of magnesium, FSH, testosterone, and glutathione and significantly decreased the concentrations of pro-oxidants, such as malondialdehyde. Scott et al. reported that selenium supplementation at a dose of 100 μg/day for 3 months significantly increased sperm motility in infertile men (118). These findings suggest that selenium potentially influences sperm quality and motility and male fertility.

### Manganese

Manganese plays a significant role in human reproductive function. It protects against formaldehyde-induced sperm parameter and testis structure damages in mice (119). Manganese supplementation can maintain thiol concentrations by reducing oxidative stress in human sperm and can enhance the quality and motility of sperm by activating adenylate cyclase activity (120). Decreased manganese concentrations in seminal plasma have been reported to produce adverse effects on semen volume and normal sperm morphology. Guiying reported that high manganese concentrations are associated with erectile dysfunction (121). Another study of infertile men showed that high manganese concentrations in seminal plasma are significantly associated with an increased risk of decreased sperm motility and count (122). High concentrations in seminal plasma are related to increased sperm survival rate and progressive motility rate and abnormal percentage of sperm count (123). These findings suggest that manganese is an essential element and that low concentrations regulate normal function of the human reproductive organs, sperm motility, and fertilization, whereas high concentrations adversely affect human sperm.

Manganese is an essential nutrient for normal human development and activates genes responsible for the production of GnRH (124). Rotter et al. found that the testosterone concentration is negatively correlated with the free androgen index and that an elevated manganese concentration is inversely associated with testosterone production (125). Exposure to manganese may affect the function of the male reproductive system, resulting in increased spontaneous abortion rates, estrogenic hyperactivity, impotence, and infertility among the wives of workers (126). Wang et al. found that long-term exposure to low concentrations of manganese decreased the concentrations of serum PRL, LH, and TSH in 41 welders (127). High serum manganese concentrations in female workers yielded a variety of effects, including increased activity of DNA oxidation products, increased incidence and variability of chromosome polymorphisms characterized by somatic genetic instability, and sex hormone imbalance (128). The manganese exposure level ranged from 0.56 to 34.25 mg/L and the average concentration was 15.92 ± 8.49 mg/L among 84 male workers and 92 referents. GnRH and LH concentrations increased significantly, while the testosterone concentration decreased significantly in the manganese-exposed group. This finding supports the significant positive correlation between urinary manganese and GnRH and LH concentrations and the significant negative correlation between urinary manganese and TSTO concentrations. Ultimately, the manganese-exposed group showed decreased sperm progressive motility and total motility.

### Copper

Copper is an essential element for many metalloenzymes involved in energy or antioxidant metabolism and protects sperm cells from oxidative damage. However, its function depends on the plasma concentration. High copper concentrations are harmful to spermatozoa function, sperm quality, sperm motility, and mitochondrial activity (129). One study found that the seminal plasma copper concentration had a positive effect on sperm parameters, including volume and motility (130). The main function of this element is to participate in sperm motility and to act on the hypothalamus-derived progonadoliberin-1 to control the release of LH. High copper concentrations (≥100 μg/mL) are harmful to sperm motility and morphology and DNA (131).

### Strontium

Strontium is a silvery metal that naturally occurs as a non-radioactive element. It is found in rocks, soil, dust, coal, and oil. About 99% of the strontium in the human body is concentrated in the bones. Strontium can exist in two oxidation states, namely 0 and +2. The isotopic form of strontium is used as a tracer in geological processes and as an indicator of provenance in archaeological contexts. Strontium has several biomedical applications, including in medicine and dietary supplements. Strontium plays an important role in reproductive health, and exposure to high concentrations is associated with low serum testosterone concentrations and an increased risk of primary male infertility (132). Studies have shown that strontium can improve animal and human sperm motility, capacitation, and the acrosome reaction (133). Clinical studies have shown that the addition of strontium to culture media can activate oocytes, thus improving fertilization and clinical pregnancy rates. Miao et al. proposed that the concentration of strontium in urine was positively correlated with sperm quality (134).

### Molybdenum

Molybdenum is an essential trace mineral in animals and humans and is part of more than 50 active enzymes. It is found in foods such as milk, cheese, cereal grains, legumes, nuts, leafy vegetables, and organ meats. Molybdenum is most commonly used for molybdenum deficiency.

Molybdenum occurs in the lithosphere at an average abundance of 1.2 mg/kg and represents one of the scarcest trace elements in biological systems and it exist predominantly in the form of the oxyanion molybdate, which serves as an essential micronutrient in all kingdoms of life. A study showed that moderate doses (25 mg/L) of molybdenum can modulate the epididymal index, sperm motility, and sperm count, whereas high doses (≥ 100 mg/L) can modulate testicular oxidative stress in a complex manner, causing negative effects on sperm (135). It promotes the normal function of cells by catalyzing hydroxylation, oxygen atom transfer, and other redox reactions (136). In a previous study, researchers investigated the effects of different concentrations of molybdenum on sperm parameters and testicular oxidative stress in adult ICR mice. A medium dose of molybdenum (25 mg/L) enhanced sperm parameters (epididymal index, sperm motility, sperm number, and morphology), whereas a high dose (≥ 100 mg/L) had no significant effect on such parameters (137).

Magnesium, calcium, copper, and manganese play significant roles in spermatogenesis and sperm motility. Calcium affects the motility, overactivation, surrender, and acrosome reaction of sperm, resulting in sperm penetration of oocytes. Copper is related to the normal function of sperm, whereas manganese affects sperm motility and fertilization (138). Urinary concentrations of molybdenum are directly proportional to the sperm concentration and total count. Researchers have demonstrated the potential importance of molybdenum in improving human semen quality. While exposure to cadmium, lead, and mercury adversely affects semen quality (139). However, exposure to certain essential elements is beneficial for semen quality because these elements are involved in maintaining normal spermatogenesis and sperm maturation. As a rich alkaline earth element, non-radioactive strontium is abundant in soil and water, as well as in cereals, vegetables, seafood, and other foods.

## Effect of nanoparticles/nanometals on spermatogenesis and male infertility

Metal nanoparticles (NPs), also called nanometals, are small particles with a relatively large surface area and a diameter between 1 and 100 nm. Due to recent advancements in nanoscience and nanotechnology, there has been great interest in metal NP applications, including in the area of reproductive biology. For example, cerium oxide (CeO2) NPs, which are able to store oxygen, act as ROS scavengers and protect sperm cell viability during cooling, and supplementting semen extender with CeO2 NPs improves sperm motility and velocity (140). Subcutaneous administration of titanium dioxide (TiO_2_) NPs at a dose of 400 μg to pregnant mice reduces the number of Sertoli cells, alters testicular morphology, disrupt seminiferous tubules, and significantly decreases daily sperm production (DSP) (141).

Zinc nano-complexes improved the functionality of sperm plasma membranes in a dose dependent manner and increase sperm count and improve sperm characteristics by protecting against oxidative stress (142, 143). Conversely, NPs of silver, TiO_2_, zinc oxide (ZnO), copper, and nickel induced oxidative stress in male reproductive organs (144–147). Olugbodi et al. reported that sub-dermal administration of AgNPs into male rats for 7 and 28 days produced histological abnormalities in the testis and alter the number of sperm, sperm motility, and testosterone, LH and FSH levels (148). A study found that intragastric exposure to TiO2 NPs induced immunological dysfunction in the mouse testis (149). Oral administration of AgNPs to Wistar rats reduced the number of sperm (150, 151). Exposure to carbon black NPs impaired the reproductive system of male offspring, including a reduction in the number of sperm in F1 males (152, 153). Oral administration of ZnO NPs to male mice significantly and dose-dependently reduced the body weight and increased the relative testicular weight (154). An *in vivo* study using mice intra peritoneally injected with iron oxide NPs caused histopathological changes such as sloughing, detachment of germ cells, and vacuolisation in seminiferous tubules of the testes (155). Similarly, exposure of adult male albino rats to TiO_2_ NPs significantly increased the thickness of interstitial spaces, induced blood vessels congestions, and promoted detachment of the germinal epithelium from the basement membrane in the seminiferous tubules (156). Treatment of with both aluminum oxide (Al_2_O_3_) NPs and ZnO NPs severely damaged seminiferous tubules and the basement membrane (157). ZnO NPs administered to albino rats induced histological abnormalities including disorganization, vacuolation, and detachment of germ cells in testicular tissues (158). Intragastric administration of TiO_2_ NPs at doses of 10, 50 and 100 mg/kg body weight induced reproductive toxicity in male mice, increased sperm malformation, and decreased the number of germ cells. Another study found that exposure of TiO_2_ (159). Xia et al. reported that curcumin functionalised NPs affected the proliferation of testicular cell lines and also causing an acute injury to the testicular functions in male mice (160).

## Other causes of infertility in men

Infertility can also caused by other issued, including problems with testicular sperm production, no sperm production, blockage or absence of the duct to transfer sperm from the testes, varicocele, hormonal imbalance, lack of sperm mobility or function, previous injuries, health factors, and ejaculation disorders.

## Conclusions and future perspectives

Male reproduction and fertility are necessary for the production of mature, healthy sperm. Proper nutrition is essential for spermatogenesis, good sperm motility and quality, and the development of Sertoli and interstitial cells. Some nutrients are involved in capacitation, hyperactivation, and the acrosome reaction. Trace elements are essential for sperm production and quality and could affect male fertility in distinct ways. Abnormal or insufficient concentrations of certain elements can cause issues during human reproduction, embryogenesis, and pregnancy. Human semen contains various micronutrients that are necessary for normal spermatogenesis and sperm maturation, motility, and capacitation. Reduced concentrations of trace elements can potentially affect sperm quality and cause infertility. Increased or decreased concentrations of trace elements can lead to sperm parameters that are thought to be the main cause of idiopathic male infertility. Environmental factors also play an important role in male fertility. Adverse environmental factors can lead to poor semen quality, an increase in the sperm DNA fragment index, and mitochondrial dysfunction, all of which can lead to male infertility. Increased EDC concentrations cause male reproductive disorders and infertility, eventually leading to a decline in male reproductive health.

However, the harmful effects of excessive trace elements are still not completely understood. Further, the molecular mechanisms underlying the positive and negative effects of many chemical and trace elements involved in male fertility are not clear. Future research should focus on identifying the key concentrations and mechanisms of these elements in relation to promoting or stopping sperm production and motility. It is necessary to investigate the complex relationships between chemical elements, enzymatic and non-enzymatic mechanisms, and other proteins and/or biomolecules. Determination of the total seminal concentration of minerals in fertile and infertile men is necessary to determine the sperm fertilization potential. In addition, it is important to determine the standards and optimal dietary nutrient concentrations required to achieve the best testicular growth, spermatogenesis, and semen quality outcomes. Additional studies on the sufficient amount of trace minerals needed for mammalian fertility could provide a solid foundation to explore the best combination of chemical elements and appropriate doses for the nutritional prevention or treatment of male infertility are needed. Future research should also focus on large consortia of well-funded organizations, including research institutes in different regions of the world. This infrastructure could allow researchers to play unbiased genomics methods and the relevant functional verification analyses on large patient cohorts. Birth cohort studies are also important to address the effects of maternal EDC exposure on semen quality and reproductive hormone concentrations.

## Author contributions

X-FZ and SG designed the study and proof read this manuscript. H-HC, YZ, and P-YD prepared this review. All authors contributed to the article and approved the submitted version.
